# Elderly: health services and human resources requirements in Portugal

**DOI:** 10.1080/07853890.2021.1896551

**Published:** 2021-09-28

**Authors:** Carla Leão

**Affiliations:** a Escola Superior de Saúde Atlântica, Barcarena-Oeiras, Portugal; b Instituto Português de Relações Internacionais IPRI-NOVA, Lisboa, Portugal

## Abstract

**Introduction:**

Portugal with the demographic aging process increased the number of elderly people. The economic global crisis printed restrictions in the National Health Service, with centralisation at the local level, distancing health services and human resources from the elderly population. This scenario gains more serious proportions because the current elderly population lives principally alone or accompanied by another elderly; has a low level of education; is economically disadvantaged; lives with chronic pathology installed. In this context, local health services and human resources are essential [1,2]. Accordingly, we put the guiding question: What adjustments should be made in health services and human resources as the Portuguese population ages, specifically at the level of primary health care, rehabilitation units, continued care and palliative units?

**Materials and methods:**

We questioned a panel of 44 experts made up of Ministers; Secretaries of State; public bodies with health functions; Orders and Associations of health professionals; Hospital Technical Commissions; public health agencies; Academics; Specialists. We used the Delphi method. The elaboration of the questionnaire went through a phase of construction and a phase of validation using a similar panel of experts. The final questionnaire is composed of 55 questions about several dimensions related to the binomial elderly/health and specifically about the dimensions: proximity; urban and rural regions; health services response as primary health care, rehabilitation units, continued care and palliative units. The questionnaire was submitted to one round because a consistent consensus was reached [1].

**Results:**

The panel answered that: the number of elderlies is an important factor to consider on health system political decisions making process; there is a need to adapt the health professions specialties; the existing services and human resources do not respond to the needs, because current services do not fit the characteristics of the current elderly; the biggest adequacy problems are located at the interior and rural level but the urban areas are also not well; the proximity of health services and primary health care are essential for the promotion and maintenance of the health and quality of life of the elderly; the units of continuous care, palliative care, and rehabilitation care, do not respond to the needs and should be multiplied in the national territory; deliberate the importance of institutions' creation that provides home health care throughout the country, to keep the elderly in their homes and close to their families and their environment; the investment in health should be focussed to the human resources and towards primary health care followed by rehabilitation care, continued and palliative care [1]

**Discussion and conclusions:**

The health services and human resources at the level of primary health care, rehabilitation care, continued and palliative care, should multiply in the national territory to increase proximity to the elderly. Specifically, primary care and rehabilitation units, with the necessary human resources, physiotherapists are implicit, to achieve excellence in elderly health and quality of life [1].
